# Continuous quality improvement (CQI) in addiction treatment settings: design and intervention protocol of a group randomized pilot study

**DOI:** 10.1186/1940-0640-9-4

**Published:** 2014-01-28

**Authors:** Sarah B Hunter, Allison J Ober, Susan M Paddock, Priscillia E Hunt, Deborah Levan

**Affiliations:** 1 Drug Policy Research Center, RAND, 1776 Main Street, Santa Monica, CA 90407-2138, USA; 2 Behavioral Health Services, Inc, RAND, 1776 Main Street, Santa Monica, CA 90407-2138, USA

**Keywords:** Continuous quality improvement, Group randomized controlled pilot trial, Stepped wedge design, Costs, Sustainment, Feasibility

## Abstract

**Background:**

Few studies have designed and tested the use of continuous quality improvement approaches in community based substance use treatment settings. Little is known about the feasibility, costs, efficacy, and sustainment of such approaches in these settings.

**Methods/Design:**

A group-randomized trial using a modified stepped wedge design is being used. In the first phase of the study, eight programs, stratified by modality (residential, outpatient) are being randomly assigned to the intervention or control condition. In the second phase, the initially assigned control programs are receiving the intervention to gain additional information about feasibility while sustainment is being studied among the programs initially assigned to the intervention.

**Discussion:**

By using this design in a pilot study, we help inform the field about the feasibility, costs, efficacy and sustainment of the intervention. Determining information at the pilot stage about costs and sustainment provides value for designing future studies and implementation strategies with the goal to reduce the time between intervention development and translation to real world practice settings.

## Background

Substance use is a significant public health problem, with an estimated 8.9% of the U.S. population needing treatment [1] and numerous social and financial costs [2], yet improvement in the quality of care for substance use disorders (SUDs) lags behind that of general health care [3]. In 2006, the Institute of Medicine (IOM) recommended a multi-faceted strategy for improving SUD care which included support for instituting quality improvement practices and increasing the use of evidence-based practices in SUD treatment centers [3]. However, to date the literature on effective implementation of quality improvement practices in SUD treatment centers remains small [4-8]. Moreover, there is limited information about CQI costs and sustainment [9-11] in these settings.

### Continuous Quality Improvement (CQI)

One potentially promising method for improving evidence-based practice delivery and quality of SUD treatment services is Continuous Quality Improvement (CQI), “a planned approach to transform organizations by evaluating and improving systems to achieve better outcomes” [12]. CQI is a concept that initially took root in the U.S. manufacturing industry in the 1920’s to improve quality and productivity [12]; now the same methods are being used to improve the quality of health care [13]. CQI involves the systematic assessment of program implementation and short-term outcomes in order to improve service delivery and long-term outcomes. CQI differs from traditional program evaluation approaches in that it involves an iterative cycle of monitoring performance, identifying problems and potential solutions, and implementing changes, as well as the involvement of frontline and other staff in the improvement process. Several studies have successfully applied CQI to the health care domain and provide evidence that CQI can be used to improve patient outcomes [14-17]; a small but growing literature suggests that CQI is feasible in SUD treatment settings [4-6,8].

Available research suggests that CQI can be successfully used to address short-term process improvements in SUD treatment settings, such as reducing wait times, increasing admissions, enhancing retention, and decreasing no-shows [4,10,18]. However, prior research is limited in scope in that it has focused primarily on process improvements rather than organizational and client outcomes, and it has yet to demonstrate evidence of long-term sustainment past the implementation during the research trial [9,11]. By sustainment, we refer to the continued use of CQI past implementation during the research trial [19]. In contrast, sustainability refers to a characteristic of an intervention that supports its continued use in practice [20]. Further, previous approaches have used a top-down approach, required extensive data tracking, and specifically focused on applying and measuring the impact of pre-specified process improvements selected by researchers [9,18,21]. In contrast, borrowing from traditional quality approaches that engage multiple levels within an organization to plan the process improvement [22], the CQI approach utilized in this study has not been well studied in addiction treatment settings. More specifically, a key difference between the CQI approach utilized in this study and previous QI initiatives tested in addiction settings [21,23] is that this CQI approach relies on treatment staff to determine the area for improvement rather than pre-determined process changes (e.g., reducing waiting time).

### Organizational change and adoption of new practices

Certain key factors contribute to the adoption and sustainment of new practices within organizational settings. These include support and leadership commitment [24]; the input of local stakeholders in the selection of organizational priorities for change [25-27]; resources, including money, materials, and access to expertise [26]; and ongoing supervision or technical assistance [23,28-30]. To facilitate adoption of new practices with organizations, interventions should be perceived as compatible with existing work practices, advantageous over similar practices, relatively easy to use, and to have demonstrable results [31]. Adoption can be further enhanced through communication networks and partnerships and through opinion leaders [32]. Strategies to enhance adoption, implementation and sustainment of new practices include increasing self-efficacy, confidence, and expectancies among staff about implementation through tools such as training and modeling and through provision of technical assistance and resources [32]. Organizational climate, such as staff cohesion, presence of opinion leaders, and openness to change contribute to an organization’s readiness to change, and thus also contribute to the adoption and sustainment of new interventions [31,33].

### The present study

In light of the utilization lag of CQI in SUD treatment settings, the paucity of effectiveness, sustainment, and cost information, and the lack of CQI interventions designed to specifically meet the needs of SUD treatment settings, the present study is examining the feasibility, preliminary efficacy, costs, and sustainment of implementing a newly designed collaborative, participatory CQI intervention [8,34]. The intervention includes a CQI toolkit, training, and ongoing onsite technical assistance. The CQI application uses data already being collected within an organization to help staff identify relevant areas for improvement; solicits input and guidance from program leadership; and provides ongoing access to a CQI expert for technical assistance. Further, the study’s modified stepped wedge design allows for the assessment of feasibility and sustainment during the pilot phase, thus maximizing resources and streamlining the movement of the intervention from research to practice. In a traditional stepped-wedge design, the trial is staged over several time periods [35-37]; in the present study, we have two study phases. A stepped wedge design typically is used when it is believed that the intervention will do more good than harm [38] and/or when there are practical or financial constraints to conducting the intervention all at once [35]. In the present study, we instituted this design to maximize the information learned from the project. During the first phase, we collect information on both feasibility (among the group assigned to receive the intervention) and on efficacy, by comparing outcomes between programs assigned to CQI (Cohort 1) and the control (Cohort 2) sites. In the second phase of the study, we continue to collect information about intervention feasibility by assigning the Cohort 2 sites to receive the CQI intervention while also monitoring CQI sustainment among Cohort 1 sites. This article describes the unique design, intervention protocol, and evaluation approach of this study.

## Methods/Design

### Study site and participants

The study site is a non-profit SUD treatment provider in Los Angeles County that receives a mix of public and private funding. Nonprofit providers represent the largest proportion (61%) of SUD treatment programs nationally (as compared to for-profit [27%] and public [12%] programs) [39]. Consistent with recommendations for a collaborative approach to organizational change and to CQI, the Director of Quality Assurance of the provider organization is a co-Principal Investigator on the study.

We selected eight of the organization’s SUD treatment programs to participate in the study. The eight programs represent typical publicly funded addiction treatment programs in Los Angeles County. We included four residential and four outpatient programs that are relatively close in size and budget. The programs each serve on average 197 (residential) or 320 (outpatient) diverse clients (i.e., 60% male; 26% White, 29% African American, 40% Hispanic, and 5% Asian and/or other) annually. Average length of stay ranges from 85 to 115 days in residential and outpatient treatment, respectively.

Although assignment to the CQI intervention is at the program level, participants at multiple levels participate in intervention activities. Specifically, onsite meetings with clinical and administrative staff at each of the participating programs are planned after random assignment so that all clinical and administrative staff are directly informed about the project. In addition, one supervisor and one member from the clinical team from each program assigned to the intervention are asked to attend monthly CQI meetings held at the organization’s headquarters with the other assigned program staff. The staff that attend these monthly CQI meetings are responsible for bringing information back to their respective programs. Other clinical and administrative staff at the sites are involved in the evaluation component of the study.

### Study design

**Figure 1:**
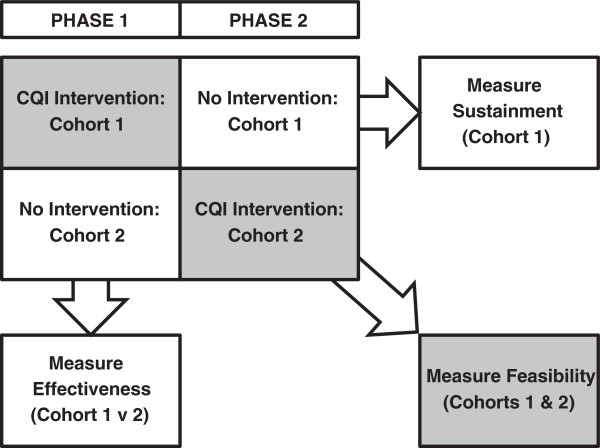
Study Design.

The study is a group-randomized trial (GRT). Eight programs, stratified by modality (residential or outpatient), are being randomly assigned to receive the intervention either during the first study phase (these programs comprise ‘Cohort 1’) or the second (Cohort 2) (see Figure 1).

### Study aims and hypotheses

The specific aims of this study are to 1) conduct a randomized pilot study to assess the extent to which the CQI intervention leads to process and outcome improvements in the organization, and 2) investigate implementation feasibility across the organization, including the a) processes and extent of implementation among staff, b) cost of conducting CQI, and c) sustainment of the CQI process within the programs over time. We hypothesize that clients attending the intervention programs will stay in treatment longer, achieve higher levels of treatment satisfaction, have higher rates of positive treatment compliance, and achieve higher functioning compared to clients attending control programs. We expect that staff in programs participating in the CQI intervention will demonstrate improvements in job morale, job satisfaction, and attitudes towards evidence-based practices compared with staff assigned to programs in the control condition. We also hypothesize that intervention programs would experience lower staff attrition than programs assigned to the control condition. These hypotheses are developmental and exploratory, consistent with the grant mechanism that supports the work (i.e., NIH Clinical Trial Planning Grant Program, R34).

With regard to implementation feasibility (Aim 2), we hypothesize that programs that report a greater number of facilitators than barriers to CQI implementation will achieve higher levels of CQI implementation; that higher ratings on perceptions of key elements of diffusion of innovations (i.e., relative advantage, compatibility with current work practices, ease of use, and observability of benefits [31]) will lead to greater CQI implementation; that organizational readiness to change will correlate with higher levels of CQI implementation; and that programs that engage in a greater number of CQI activities will experience higher overall levels of CQI implementation. In our evaluation of sustainment we expect that programs that show higher levels of CQI activity during the intervention will sustain more components than programs with lower levels of activity and that more positive perceptions of CQI and organizational readiness to change at the end of the intervention will predict CQI sustainment.

### The RAND CQI intervention

The CQI intervention was designed to assist program staff in utilizing the Plan-Do-Study-Act (PDSA) approach. The PDSA approach was first developed by Walter Shewhart, later refined by W. Edwards Deming [40], and recently made popular in medical service settings by the Institute for Healthcare Improvement (IHI). We also utilized materials from RAND’s work in improving practice guideline implementation in the U.S. Army’s medical systems [41] and RAND’s collaborative quality improvement projects with Phoenix House, a national addiction treatment organization (R01 DA14969, Wenzel, PI) to help inform development of the intervention. To adapt the previous CQI approaches for community-based care, where outputs are services delivered rather than a tangible manufactured product or a specific procedure (like hospital-based care), changes in the terminology and measures were made. We developed and piloted this CQI intervention in conjunction with staff from a different SUD prevention and treatment organization [8].

PDSA is an approach in which improvement strategies are identified and tested using a small pre-post pilot study approach. Results from small, pre-post pilots are used to determine whether the change should be incorporated into a program’s standard practices. In the RAND CQI intervention, CQI activities are framed around regularly scheduled meetings during which staff develop “CQI Actions” or specific improvement plans. These plans are based on a systematic assessment of program processes and on outcome data. The intervention also incorporates an empowerment evaluation approach [42], where staff, with assistance from the PIs and organizational leadership, systematically assess their own programs and develop CQI Actions. Staff are coached on utilizing existing process and outcome data to identify areas for improvement which are then vetted within the larger organization. Process and outcome data refer to information related to the admission and intake process (e.g., waiting list and admission procedures), service delivery (e.g., length of stay, type of treatment received), and treatment impact (e.g., client discharge status, client functioning after treatment ends). Following the identification and vetting of a CQI Action, staff implement the CQI Action, examine its impact, and decide on next steps. During this period, programs received technical assistance to help guide them through the PDSA cycle. Program leaders are expected to meet monthly throughout the process to discuss progress with other participating program staff.

### Implementation approach

We used the Simpson Transfer Model (STM) [43] as a guide to plan implementation of the RAND CQI intervention. The STM involves four stages of innovation transfer: (1) Exposure, or introduction and training in the innovation; (2) Adoption, which refers to an intention to try the innovation through program leadership decisions and subsequent support; (3) Implementation, or exploratory use of the innovation, with customization by the organization; and (4) Practice, which refers to the routine use of and performance feedback on the innovation. The STM incorporates staff readiness to change [44] and Rogers’ Diffusion of Innovation theory [31] at both the individual (staff) and organizational levels. For example, crucial to moving from the exposure to implementation stage are resources provided by the institution (e.g., training, leadership), organizational characteristics such as “climate for change” (e.g., staff cohesion, presence of opinion leaders, openness to change), and staff perceptions of the innovations (e.g., complexity, benefit, and observability). Consistent with this idea, the organizational change literature suggests that several factors are needed to put a new business practice into place such as support and leadership commitment [24], stakeholder involvement [25], resources including money and materials, and access to expertise [26]. More recently, an emerging literature regarding the diffusion of evidence-based treatment suggests that training in a new intervention must be supported by ongoing supervision or technical assistance in order for new behaviors to be adopted and sustained [28,29].

The implementation of the RAND CQI intervention incorporated these four stages of STM. Regarding *Stages 1 and 2: Exposure and Adoption*, the STM predicts that adoption and exposure of a new innovation or intervention are most likely to occur when facilitated by organizational units that control resources and policies. Therefore, we began the exposure and adoption stages at the proposal phase by obtaining buy-in from the Quality Assurance Director (Levan) and the Chief Executive Officer and Chief Operating Officer to ensure that the organization provided support for training in the intervention to clinical staff. Although a key element of CQI is not intended to be a top-down approach, gaining the support of key leadership is consistent with implementation theories put forth by Aarons et al. [19] and Fixsen et al. [26] that argue that leadership support is critical for successful implementation. Upon receipt of funding, the co-Principal Investigators (Hunter and Levan) started to plan the intervention’s launch in a way that would increase exposure across the organization and start the implementation phase. Following baseline data collection and randomization, the RAND CQI intervention begins with regularly scheduled monthly meetings to introduce key program staff (i.e., program and/or clinical director and one member of the clinical staff assigned to the intervention) to the PDSA cycle. The organization is being compensated for staff participation in these meetings and the meetings are scheduled at a time when staff did not have existing commitments to enhance exposure.

The *Stage 3 Implementation* phase is characterized by exploratory use of the intervention, supported by tools and assistance. The monthly CQI meetings incorporate active learning strategies that help promote retention and understanding of key concepts [45,46]. The meetings are also designed to facilitate buy-in and accountability across the organization. The first three monthly CQI meetings provide guidance to program staff on the “Plan” phase of PDSA. More specifically, program staff are asked to assess different parts of their program including referral, intake, service delivery, and outcomes to identify strengths and weaknesses that may be targeted for improvement. During this process, staff are also asked to link process and outcome goals to measurable objectives, which are documented in worksheets included in the CQI toolkit. Following this data review and planning process, staff are asked to identify and document a “CQI Action” (i.e., a small improvement plan) and document methods to study its impact (i.e., the “Do” and “Study” phases of PDSA). Accompanying worksheets in the CQI toolkit are completed to document the CQI Action, along with specific tasks and timelines associated with it. This work occurs in a group setting with assistance from the PIs and followed by feedback by senior organizational leadership. Next, key program staff continue to meet monthly to give updates and discuss any challenges with the PDSA approach or implementing their CQI Action. With the presence of the organizational leadership at these meetings, problem-solving that is contingent upon agency resources or other leadership decisions are addressed. Following the completion of the PDSA cycle, staff are guided by the co-PIs to refine the first CQI Action or develop a new CQI Action.

In *Stage 4 Practice* ***,*** performance monitoring and feedback, which are critical to implementing innovations, are emphasized. The monthly meetings and contact via email and phone between the participating staff and the Implementation Team Leads (co-PIs, Hunter and Levan) are designed to provide the coaching and feedback needed to build and sustain competency [47]. Throughout the practice phase, staff are asked to complete CQI toolkit worksheets and share them with the Implementation Team Leads to assist in monitoring progress and provide opportunities for feedback.

### Measures and procedures

#### Process and outcome measures

To measure differences in process and outcome improvements between the intervention (Cohort 1) and control (Cohort 2) groups, we are examining: (1) staff job morale, (2) job satisfaction, (3) attitudes toward adopting evidence-based practices, and (4) attrition. Client outcomes are (1) length of stay in treatment, (2) satisfaction with treatment, (3) clinical status at discharge, and (4) post-treatment functioning.

To collect staff data, we are administering a web-based survey to staff at six month intervals, starting with a baseline survey that is being administered prior to study assignment. We are using the following measures to obtain data on staff outcomes: *Morale.* The job morale questionnaire consists of nine statements taken from the emotional expression subscale of the Maslach Burnout Inventory [48]. The inventory assesses feelings of emotionally overextension and work exhaustion and has high reliability (α = .90). *Job Satisfaction*. The job satisfaction scale consists of six items related to satisfaction with different job aspects [49]. The scale is part of Texas Christian University’s Survey of Organizational Functioning (TCU SOF) instrument [49] which measures 4 domains: motivation for change, institutional resources, staff attributes, and organizational climate. *Evidence-Based Practice Attitudes.* To measure attitudes towards evidence-based practices, we are using the Evidence-Based Practice Attitude Scale (EBPAS) [50], a 15-item survey that assesses providers’ attitudes about adopting EBPs; reported reliability among mental health providers is good (α = .79). *Staff Attrition*. We are monitoring staff employment rates at each of the programs to track attrition rates throughout the study. The participating organization keeps administrative records on employment.

Our measures of client outcomes are as follows: *Length of Stay.* Client length of stay refers to the time from treatment admission to the date of last service. The organization maintains these data on all clients. *Satisfaction*. The client satisfaction survey contains seven yes/no survey items, as follows: (1) Was it easy to make an appointment for assessment or admission; (2) Is the staff polite and professional when you call on the phone; (3) Are you treated with dignity and respect; (4) Is the staff sensitive to you and your culture; (5) Are the groups informative to you; (6) Did the staff ask you about your strengths, needs, abilities, and preferences; and (7) Did you take part in your treatment planning. In addition, clients “grade” 4 aspects of their treatment on a five-point scale (Very good; Good, Average, Poor, Very Poor): the admission experience, counselor, facility/accommodations, and treatment experience. *Clinical Status at Discharge.* Participants are coded by their primary treatment counselor as discharged with either positive or negative compliance. Positive compliance is coded as: completed treatment-referred/transferred, completed treatment-not referred/transferred, left before completion with satisfactory progress-referred/transferred, and left before completion with satisfactory progress-not referred/transferred. Negative compliance is coded as: left before completion with unsatisfactory progress–referred/transferred, left before completion with unsatisfactory progress–not referred/transferred. *Client Functioning Post-Treatment* is measured through a self-report survey that asks about the past 60 days: frequency of substance use (response options: daily, 3-6 times/week, 1-2 times/week, 1-3 times/month, no use in past 30, no use in past 60 days) and whether client (yes/no): a) has been arrested, b) is employed or in school or job training, c) has received emergency medical care, or d) is attending self-help groups. All client outcome data rely on existing procedures that the participating organization utilizes to monitor its programs. This choice was primarily based on the decision to build an intervention that could be sustainable without the support of a research trial.

The organization tracks client length of stay in an administrative database. Client satisfaction data are collected at all programs during a quarterly “Client Satisfaction Week” in which the organization’s Director of Quality Assurance directs program directors to collect satisfaction surveys on all clients in attendance during that week. Discharge status is entered into the organization’s internal database when clients exit the program (i.e., after completion of the program, transfer to another program, when they leave a residential program, or 30 days without the receipt of services in outpatient programs). Client functioning post-treatment is assessed at 60 and 180 days following discharge. Clinical and non-clinical staff from the programs phone clients to complete the survey at that time by phone.

#### Implementation feasibility measures

To measure feasibility, we are examining the processes and extent of implementation, implementation costs, and sustainment. We collect process and extent data in three ways: (1) through semiannual semi-structured interviews with program staff who have been involved in the CQI meetings (i.e., program supervisors and clinicians); and (2) through surveys with the entire administrative and clinical staff that measure perceptions of CQI and organizational functioning, including readiness to change.

In the semi-structured interviews with key program staff, we are collecting information on the extent and nature of implementation of the PDSA cycle and on facilitators and barriers to implementation. We examine the extent of PDSA activity using both open- and close-ended interview questions based on an interview protocol developed to measure innovation use (Levels of Innovation Use (LOU) interview by Hall & Hord [51]). We are examining the nature of the CQI Actions through open-ended questions, and progress within the PDSA cycle through close-ended questions. To determine whether programs implement the Plan-Do-Study-Act phases, we are coding how many of the stages of the PDSA cycle (0-4) and how many PDSA cycles staff achieved during the intervention period. To examine facilitators and barriers to implementation, we are using items adapted from Scheier et al. [52] and Grol and Wensing’s [53] standard items for evaluating barriers and facilitators.

Through web-based staff surveys conducted every six months, we are evaluating perceptions of CQI and organizational readiness to change. To measure perceptions of CQI, we adapted Moore and Benbasat’s [54] instrument that measures Roger’s elements of diffusion. Sub-scales of this instrument include relative advantage over usual practices (5 items; α = 0.90), the complexity/ease of use (4 items; α = 0.84), compatibility (3 items; α = 0.86), observability/demonstrability (4 items; α = 0.79), and trialability of the innovation (2 items; α = 0.71). To measure organizational readiness to change we are using Texas Christian University’s Survey of Organizational Functioning (TCU SOF) instrument [49] which measures 4 domains: motivation for change, institutional resources, staff attributes, and organizational climate.

##### *CQI Costs*

We are capturing the direct and indirect costs of CQI activities using modified versions of the standardized cost instrument for drug treatment services, the Substance Abuse Services Cost Analysis Program (SASCAP™) Labor and Cost Modules. As an innovation in costing SUD treatment programs, we also are attempting to capture opportunity costs, that is, the activities forfeited to conduct CQI activities. Specifically, our costing strategy captures the following: Direct cost – the direct expense outlay to accomplish given CQI activities; Indirect cost – the amount of time, effort and other organizational resources spent, but not as a direct cash outlay, for CQI activities; and the Opportunity cost – the net benefit of the activities foregone. The costing approach is based on the principle of Activity-Based Costing (ABC), using the data collection tools and interviews with staff members to define staff activities involved in the additional CQI intervention activities, and asking staff how they allocate their time to these activities and then in turn to products and services.

We modified the SASCAP Labor- and Cost-Modules for non-methadone outpatient services [55,56] to include aspects of residential services for those sites. Furthermore, unlike the focus of SASCAP, which is on the overall cost of one treatment program, the CQI project involves a large range of activities of varying time expended. As such, we are implementing a monthly cost questionnaire to more accurately attribute time spent on CQI activities and materials purchased rather than relying on reports at less frequent intervals.

The annual cost data collection tool includes the following: General information about the clients served including census, capacity, and length of stay; information about average salaries and number of employees, by category of personnel; capital expenses for the program, organized by category – including equipment, materials and infrastructure improvements (if any); and recurrent operational expenses used for client care, organized by category – including equipment, office supplies and utilities. Given the data systems in place and the need to reduce reporting burden, we designed a ‘corporate’ and ‘program’ version, each with unique questions. Facility program directors and a corporate compliance officer meet with a cost study analyst to input data for each cost line item using administrative, financial data. The annual cost questionnaire is filled out for the year prior to CQI (baseline) and during the year of CQI, allowing for analysis as to whether CQI affected other costs indirectly.

The monthly questionnaire collects: a) Information about man-hours spent by category of personnel and CQI activity; and b) Recurrent operational expenses used for CQI activities specifically, organized by category – including equipment, office supplies, and utilities. In the CQI trial, program staff are advised to keep in mind the CQI activities that they and other staff at their site perform. During each monthly CQI meeting, program staff recall those activities and fill out the questionnaire regarding time spent and materials purchased over the last month as a direct result of engaging in CQI.

##### *Sustainment*

To assess whether CQI activities are sustained, we plan to examine data collected from Cohort 1 during the Phase 1 implementation interviews and again during the Phase 2 period when the Cohort 1 programs are no longer receiving the intervention. These data will inform us about which CQI activities are sustained in Cohort 1 (if any), and about facilitators and barriers to sustainment. To collect these data, we are building upon the semi-annual implementation interview protocol in two ways. First, during the last set of Cohort 1 interviews, we are asking program staff about their plans to sustain CQI. Second, during the Cohort 2 intervention period, we are asking Cohort 1 staff whether the different components of the intervention are continuing, that is, whether regular CQI meetings are taking place.

### Analytic methods

The randomization of programs to study conditions is necessary given the CQI intervention is intended to change program-level practices. However, it is important to assess the effect of the multilevel study design on the statistical power to detect intervention effects [57]. One key component of the power calculations is the intra-cluster correlation (ICC), which is the proportion of the overall variance in the outcomes that is attributable to program membership. For a given sample size, statistical power decreases as ICC increases. We therefore present below our analytic approach for the analysis of each outcome, and note for which outcomes we expect to have sufficient statistical power to examine, based on ICC data from previous studies.

#### Intervention efficacy

The first set of analyses will focus on examining the effect of the CQI intervention on staff outcomes relative to the control condition (Aim 1). The hypotheses related to staff outcomes are that staff participating in the CQI intervention will demonstrate improvements in: a) job morale, b) job satisfaction, and c) attitudes towards EBPs as compared with staff assigned to the control condition. Improvement will be measured as the difference between pre-intervention and post-intervention (i.e., end of Phase 1) assessments. Improvement in staff-level outcomes will be modeled using linear multilevel regression modeling, controlling for baseline values of job morale (a), job satisfaction (b), and attitudes towards EBPs (c) when modeling those outcomes. The multilevel model structure will account for the similarity, or intra-cluster correlation (ICC), among staff outcomes within the program. The statistical significance of a dichotomous predictor of participating in the CQI intervention and its direction will be examined to test these hypotheses. We will also examine whether programs that participate in the CQI intervention experience lower staff attrition than programs assigned to the control condition (d). Given the sample size of programs in the study, we will use descriptive statistics to examine whether staff attrition is lower for staff participating in the CQI intervention.

For analyses of the staff-level outcomes (a-c)*,* we assume an intra-cluster correlation (ICC) of 0.040 for staff-level outcomes, which is obtained by averaging ICCs for data on similar measures from two external studies. One ICC estimate is from staff attitudes toward implementing ‘Getting To Outcomes,’ a site-level intervention that incorporates CQI and other elements to increase organizational evaluation capacity [58]. Other ICC estimates are for subscales from the Organizational Readiness for Change (ORC) instrument [49] that was implemented at four SUD treatment program sites from same organization participating in a prior RAND study [59]. We will have 80% power (alpha = 0.05, two-sided test) to detect an effect size of 0.63 standard deviations (SDs). Our analyses of staff attrition (d) will be limited to exploratory analyses given the lack of statistical power for formally testing whether CQI has an effect on attrition.

The second set of analyses will focus on client outcomes. As most client-level outcomes are reported dichotomously, we will fit for most outcomes multilevel binomial regression models to test these hypotheses. Other forms of the generalized multilevel model will be used for continuous outcomes as appropriate. The statistical significance of the dichotomous predictor of being assigned to the CQI intervention will be examined.

Table 1 summarizes the power calculations for the client-level outcomes. Column 1 provides an estimate of the intra-class correlation (ICC) from the preliminary program data; column 2 shows the base rate assumed for the comparison group on each outcome available to us in preliminary program data; and column 3 shows the percentage point difference in the outcome between the intervention versus control arm (assuming it maintained the assumed base rate) that would be detectable with 80% power (alpha = 0.05, two-sided test). The arrest rate outcome is not presented in this table, as its base rate is too low and ICC too high to yield an outcome for which we would have sufficient statistical power to detect meaningful effects.

**Table 1:** Percentage point difference detectable assuming control group base rate and intra-cluster correlations

**Outcomes**	**(1) Intra-cluster correlation**	**(2) Base rate (for control arm)**	**(3) Percentage point difference detectable**
Length of stay > 90 days	0.008	50.0%	10.7
Satisfaction:% rating treatment experience as ‘Very Good’	0.110	52.0%	24.0
% giving counselor a 'Very good’ rating	0.038	78.0%	11.4
Positive compliance	0.002	60.0%	6.6
% weekly or more frequent substance use*	0.048	10.9%	8.3
Employed or in school*	0.150	64.4%	26.0
Positive employment activities*	0.010	16.0%	7.2
Emergency medical care*	0.009	8.2%	4.2
Attended self-help groups*	0.008	75.4%	6.9

*Measured at baseline and follow-up. The power calculations reflect the assumption that the correlation between baseline and follow-up observations of the same measure is 0.7 and that the baseline measure is a covariate in the analytic model.

#### Feasibility

To examine the process and extent of CQI implementation, we will examine whether higher ratings of relative advantage, compatibility, observability and trialability, and organization readiness to change will be correlated with higher levels of CQI implementation. CQI implementation will be assessed by ratings of PDSA Activity. Ratings of innovation attributes and organizational climate will be associated with PDSA Activity. We will test this hypothesis by regressing each staff-level outcome (relative advantage, compatibility, observability and trialability, and organization readiness to change) on PDSA Activity rating at follow-up, while controlling for baseline values of these measures. The statistical significance and direction of the coefficient on the PDSA Activity will be examined to test this hypothesis. Under similar assumptions about the ICC of responses among staff within programs, we will have 80% power (alpha = 0.05) to detect whether PDSA Activity rating predicts at least 17% of the variance (e.g., R^2^ = 0.17) of each outcome. To examine whether programs that engage in a greater number of CQI activities will experience higher levels of CQI implementation, we will assess the number of CQI activities (workshop and CQI meeting attendance, worksheet completion) by each program to assess the extent of CQI activity. CQI implementation will be assessed by ratings of PDSA Activity. We will test this hypothesis by examining the correlation between the number of CQI activities completed and PDSA Activity.

#### Sustainment

To examine whether perceptions of CQI and organizational readiness to change at the end of the intervention will predict CQI sustainment, we will explore bivariate relationships between innovation attributes (i.e., perceptions of the CQI’s relative advantage, complexity, observability, and trialability) and organizational climate as assessed at the end of the intervention with sustainment at end of Phase 2, examining the correlation between the two. We will have 80% power (alpha = 0.05) to detect whether PDSA Activity rating predicts at least 31% of the variance (e.g., R^2^ = 0.31) of each outcome; given the power limitations this should be regarded as a descriptive rather than inferential analysis.

#### Costs

Since the monthly cost surveys are activity-based and the interview questions on costs will provide details for how time on CQI activities were spent (e.g., additional work, part of work already doing), we will initially describe direct costs associated with key types of CQI activities. We will then aggregate these activities and use findings from the annual cost survey and interviews to provide a description of the direct and indirect costs of implementing CQI. Given the lack of information regarding CQI costs, we view this in itself as a contribution to the literature and to practitioners. Lastly, we will conduct a standard cost analysis comparing costs between the facilities that receive the intervention and facilities that had not yet, according to the cohort schedule.

## Discussion

The present study offers a much-needed, innovative strategy for implementing CQI in SUD treatment settings. While CQI may offer ways to improve SUD treatment, we cannot assume that it can be easily transported into practice or that it will have the same impact as it has had in more traditional healthcare settings [60]. Organizations do not always adopt new practices even when they are known to improve outcomes [61]. Further, attention needs to be paid to factors both at the individual and organizational levels that impact the degree to which new practices are adopted and implemented. For example, a number of studies have shown that factors at both the individual level (e.g., training, skills, efficacy, involvement in decision making, and job satisfaction) and at the organizational level (organization size, climate, and financial resources, and active leadership support) predict successful program implementation [61-66].

Our strategy for bringing CQI into SUD treatment settings addresses many of these needs and potential transportability barriers. First, collaboration with organizational leadership and their active participation in meetings and workshops helps tailor the intervention to the needs of the organization and lends credibility to the intervention. Next, the study provides clinical staff with skills to make small improvements to enhance treatment quality and involves them in decision-making and planning activities. And finally, through hands-on skill-building meetings and ongoing technical assistance, the intervention offers an active learning environment for staff at multiple levels.

Moreover, the study offers a unique design that provides a way to maximize the collection of feasibility, costs, efficacy, and sustainment of the intervention in a pilot study. In particular, data collection instruments were designed to permit a relatively comprehensive analysis of the intervention, yet were implemented so as to reduce the burdens of data collection and performance of multiple analyses. Although we are somewhat limited by testing the intervention in a single organization, it is recommended that early stage intervention studies take place in a single organization to eliminate the potential loss of power involved in statistically adjusting for organizational differences [67]. Another potential limitation of the study is that the proposed assessments (i.e., staff and client outcomes) may not adequately reflect change as the result of a type of CQI interventions selected. That is, because program staff determine the CQI improvement strategy and it is likely to differ across programs, it is not known how well a specific intervention will be aligned with the outcomes being evaluated in the study. To address this limitation, the study captures both staff and client level changes, as CQI strategy may influence one but not the other. This is also consistent with the results from CQI practiced in traditional health care settings, where studies have shown that CQI efforts have demonstrated tangible results to both patients and providers [68]. In summary, we hope that this study design offers a template for those wishing to efficiently study intervention feasibility, cost, efficacy, and sustainment in a pilot study which may shorten the time between intervention development and translation into real world practice settings.

## Abbreviations

ABC: Activity based costing; CQI: Continuous quality improvement; EBPAS: Evidence-based practice attitude scale; GRT: Group randomized trial; ICC: Intra-cluster correlation; IOM: Institute of Medicine; NIH: National Institutes of Health; ORC: Organizational Readiness for Change; PDSA: Plan-Do-Study-Act; SASCAP: Substance Abuse Services Cost Analysis Program; SD: Standard deviation; STM: Simpson transfer model; SUD: Substance use disorder; TCU SOF: Texas Christian University’s Survey of Organizational Functioning.

## Competing interests

The authors declare that they have no competing interests.

## Authors’ contributions

SH, SP and DL conceptualized the study and obtained funding. SH has overall responsibility for the execution of the CQI intervention, data collection, analyses and reporting. DL is primarily responsible for coordination of the treatment site intervention participation and client data collection. AO contributed to the draft of the manuscript, assists with study coordination, data collection and qualitative data analyses. SP will conduct the statistical analyses. PH will perform the cost-related analyses. All authors read and approved the final manuscript.
